# A cross-species analysis of neuroanatomical covariance sex difference in humans and mice

**DOI:** 10.1101/2024.11.05.622111

**Published:** 2024-11-05

**Authors:** Linh Pham, Elisa Guma, Jacob Ellegood, Jason P. Lerch, Armin Raznahan

**Affiliations:** 1 Section on Developmental Neurogenomics, Human Genetics Branch, National Institute of Mental Health, Bethesda, 20892, Maryland; 2 Mouse Imaging Centre, Toronto, Ontario M5T 3H7, Canada; 3 Bloorview Research Institute, Holland Bloorview Kids Rehabilitation Hospital, Toronto, Ontario M4G 1R8, Canada; 4 Department of Medical Biophysics, University of Toronto, Toronto, Ontario M5G 1L7, Canada; 5 Wellcome Centre for Integrative Neuroimaging, University of Oxford, Oxford, OX3 9DU, United Kingdom; 6 South Texas Medical Scientist Training Program, University of Texas Health Science Center San Antonio, San Antonio, 78229, Texas; 7 Harvard Medical School, Boston, 02115, Massachusetts; 8 Department of Pediatrics, Lurie Center for Autism, Massachusetts General Hospital, Lexington, 02421, Massachusetts

## Abstract

Structural covariance in brain anatomy is thought to reflect inter-regional sharing of developmental influences - although this hypothesis has proved hard to causally test. Here, we use neuroimaging in humans and mice to study sex-differences in anatomical covariance - asking if regions that have developed shared sex differences in volume across species also show shared sex difference in volume covariance. This study design illuminates both the biology of sex-differences and theoretical models for anatomical covariance – benefitting from tests of inter-species convergence. We find that volumetric structural covariance is stronger in adult females compared to adult males for both wild-type mice and healthy human subjects: 98% of all comparisons with statistically significant covariance sex differences in mice are female-biased, while 76% of all such comparisons are female-biased in humans (q < 0.05). In both species, a region’s covariance and volumetric sex-biases have weak inverse relationships to each other: volumetrically male-biased regions contain more female-biased covariations, while volumetrically female-biased regions have more male-biased covariations (mice: r = −0.185, p = 0.002; humans: r = −0.189, p = 0.001). Our results identify a conserved tendency for females to show stronger neuroanatomical covariance than males, evident across species, which suggests that stronger structural covariance in females could be an evolutionarily conserved feature that is partially related to volumetric alterations through sex.

## INTRODUCTION

Structural covariance refers to the phenomenon in which variable biological structures in a population scale together across individuals. That is, if an individual has a small structure A, then they are likely to have a small structure B and vice versa. The degree of covariation between two structures is typically taken as evidence for how strongly they relate at some unmeasured levels of biology (Galton, 1888). Structural covariance relationships in adulthood have been shown to partly cohere with shared transcriptomics and connectivity between brain regions (Andrews et al., 1997; Gong et al., 2012; Lerch et al., 2006; Mechelli et al., 2005; Romero-Garcia et al., 2018; Yee et al., 2018) – potentially reflecting coordinated anatomical maturation (Alexander-Bloch et al., 2013b; Raznahan et al., 2011) via genetics, cellular patterning and experience-dependent plasticity (Alexander-Bloch et al., 2013a; Evans, 2013). However, direct causal tests of these hypotheses have been hard to achieve as they would require coordinated experimental manipulations of multiple brain regions across individuals in a longitudinal study design.

Here, we use the naturally occurring comparison between males and females of both humans and mice as a powerful window to test theoretical models for structural covariance in the brain. As an alternative to experimental manipulation of multiple brain regions, we set out to study how covariance differs under the regional developmental influences of sex. Experimental data have identified murine brain regions that show reproducible volumetric sex differences (Qiu et al., 2018) through regional action of male-specific hormonal effects that are not operative in females (Forger et al., 2004; Gorski et al., 1978; Hines et al., 1992; Roos et al., 1988; Segovia et al., 1984). These volumetric sex differences include male-biased volume in the bed nucleus of the stria terminalis (BNST), olfactory bulb, medial amygdala, and female-biased volume in the anteroventral periventricular nucleus (AVPV) (Forger et al., 2004; Hines et al., 1992; Roos et al., 1988; Segovia et al., 1984). Humans also show highly reproducible sex differences in regional brain anatomy (DeCasien et al., 2022; Liu et al., 2020) that partly cohere with those seen in mice (Guma et al., 2024) and presumably also reflect sex-biased regional brain development. If adult neuroanatomical covariances arise through an inter-regional sharing of developmental influences, then regions evidencing sex-biased developmental influences (as evidenced by sex differenced in their mean adult volume) would be predicted to also show sex-biased volume covariance. Thus, the study of sex-differences in neuroanatomical covariance can not only shed light on an understudied potential axis of sex-biased brain organization, but also provides a unique naturally occurring test for developmental models of anatomical covariance more generally. To date however, there is pronounced heterogeneity in results across those few studies that have tested for sex-biased structural covariance in humans (Mechelli et al., 2005; Persson et al., 2014; Seitz et al., 2019; Shi et al., 2023; Vijayakumar et al., 2021; Wierenga et al., 2018), without comparison to sex-differences in regional volume. To our knowledge, there are no published studies of sex-biased neuroanatomical covariance in mice.

Here, we use cross-species structural magnetic resonance imaging (sMRI) to map and compare sex-biased brain volume covariance – asking how any such sex-biases relate to sex-differences in regional brain volume. We first consider all inter-regional pairs collectively, and test for a tendency towards stronger volume covariance in one sex than the other of each species. These analyses also provide the first direct comparison for the strength of inter-regional neuroanatomical covariance in humans as compared to mice. We then score all brain regions in each species for the magnitude of their sex-biased covariance with other regions, and further resolve sets of inter-regional pairings with prominent sex-biased volume covariance. These novel maps enable us to systematically test if inter-regional variation in the strength of sex-biased volume covariance is related to interregional variance in the magnitude of sex-biased volume. Taken together our analyses provide the first comparative analysis of sex-biased neuroanatomical covariance in humans and mice. Our results inform dominant developmental theories for the emergence of structural covariance and expand our comparative understanding of sex-biased mammalian brain organization.

## METHODS

### Acquisition and processing of murine neuroimaging data

Our study includes structural MRI (sMRI) brain scans from 423 mice acquired at the Mouse Imaging Centre in Toronto. Scans were performed on the same 7T multichannel scanner with either an insert gradient (6 cm inner bore diameter magnet) or an outer gradient (30 cm diameter bore diameter magnet) (Agilent Inc., Palo Alto, CA). Mice were transcardially perfused using a standard protocol across all cohorts (Cahill et al., 2012; Lerch et al., 2011; Spring et al., 2007). Brains were kept in the skull and fixed to avoid distortions during imaging. All animal procedures were approved by the ethics committees of their originating labs and the animal care committee at The Centre for Phenogenomics (AUP-0260H) at the University of Toronto.

As the mouse data for this study was collected over 10+ years, the MRI pulse sequences were optimized over that time period to increase the scanning throughput to enable 16 mice to be scanned in one session, and/or to improve resolution and increase gray/white matter contrast in each scan (Ellegood et al., 2015; Lerch et al., 2011). The following three MRI sequences were used in overnight scans throughout the studies included here. (1) 3 brains scanned in parallel per session -- T2-weighted fast spin echo (FSE): TR = 325 ms, TE = 10 ms/echo for 6 echoes. The center of k-space is acquired on the 4th echo. Field-of-view (FOV) = 14 × 14 × 25 mm^3^. Matrix size = 432 × 432 × 780. Image resolution = 32 μm isotropic voxels. (2) 16 brains scanned in parallel per session (sequence 1) -- T2-weighted 3D FSE: TR = 2000 ms, echo train length = 6, TE_eff_ = 42 ms. FOV = 25 × 28 × 14 mm^3^, matrix size = 450 × 504 × 250. Image resolution: 56 μmm isotropic voxels. Oversampling in the phase encoding direction by a factor of 2 was applied to move ghosting artifacts from k-space discontinuity to FOV edges. FOV was cropped to 14 mm after image reconstruction. (3) 16 brains scanned in parallel per session (sequence 2) -- T2-weighted 3D FSE: TR = 350 ms, TE = 12 ms/echo for 6 echoes. Cylindrical 3D k-space acquisition. FOV = 20 × 20 × 25 mm^3^, matrix size = 504 × 504 × 630. Image resolution: 40 μmm isotropic voxels (Spencer Noakes et al., 2017). Of note, these sequences were evenly distributed between male and female mice.

Structural MRIs were registered and warped to an average study mouse template using deformation-based morphometry (Avants et al., 2009, 2011; Collins et al., 1994; Eskildsen et al., 2012; Friedel et al., 2014). Log-transformed Jacobian determinants for each voxel were calculated and used to determine voxel volume differences between individual mouse brains with the averaged brain (Chung et al., 2001). ROI volumes were calculated as the sum of volume differences for each voxel within the ROI. This process used the MAGeT algorithm (Chakravarty et al., 2013; Pipitone et al., 2014) and resulted in 336 unique brain regions from previously published atlases (Dorr et al., 2008; Qiu et al., 2018; Richards et al., 2011; Steadman et al., 2014; Ullmann et al., 2013). 255 of these regions were grey matter and included in the study.

The imaged mice consist of C57BL6J (n = 152) and C57BL6N (n = 271) wild-type controls from separate studies that each compared wild-type controls with mutations of a different autism-related risk gene (Ellegood et al., 2015). For the purposes of this study, we only included wild-type cohorts which had at least 5 male and 5 female mice surviving a quality assessment procedure to flag and remove outliers. This procedure involved an initial visual quality control was performed to ensure accurate registration and segmentation, followed by an outlier detection process.

Regional volumetric measures for the full set of 432 sMRI scans was then subjected to batch control using ComBat (*sva* library in R) to correct for variability between strain and ASD gene cohort of origin (Fortin et al., 2018, 2017; Johnson et al., 2007; Leek et al., 2022). Final animal characteristics are detailed in Table 1.

### Acquisition and processing of human neuroimaging data

This study includes 436 human sMRI brain scans from the Human Connectome Project 1200 release. Scans were obtained using an MR750 3-T (General Electric) whole-body scanner (MP-RAGE-T1: TE 2.14 ms, TR 2400 ms, flip angle = 8°, FOV 224 × 224 mm2, scan time = 7:40 min, voxel size = 0.7 mm isotropic) with a 32-channel head coil (176 continuous sagittal slices with 256 × 256 in-plane matrix and 1 mm slice thickness). Additional recruitment procedures and acquisition parameters are detailed in the original publication (Glasser et al., 2013; Van Essen et al., 2012). Information on how to obtain HCP data can be found here (https://www.humanconnectome.org/study/hcp-young-adult/document/wu-minn-hcp-consortium-restricted-data-use-terms). 1110 unique subject scans were visually inspected and removed if obvious registration and/or segmentation issues were detected. Euler numbers were also measured for each scan using the image preprocessing steps described below. Scans with FreeSurfer-estimated Euler numbers less than −217 were excluded from further analyses. From the remaining 1030 subject scans, we randomly selected one person per family, based on distinct mother ID and father ID, to yield 436 unique and unrelated subjects (Rosen et al., 2018). Final participant characteristics are detailed in Table 2.

The PreFreesurfer pipeline was used to preprocess T1-weighted structural MRI data (Glasser et al., 2013). Freesurfer 7.1.0’s (Fischl, 2012) *recon-all* and *highres* commands were used to reconstruct and parcellate the cortex at the original data resolution (Dale et al., 1999; Dale and Sereno, 1993; Desikan et al., 2006; Fischl et al., 2004a, 2002, 2001, 1999, 1998; Fischl and Dale, 2000; Han et al., 2006; Jovicich et al., 2006; Kuperberg et al., 2003; Reuter et al., 2010; Zaretskaya et al., 2018). The pipeline can be downloaded here (http://surfer.nmr.mgh.harvard.edu/). Cortical volumes were extracted using the *mri_anatomical_stats* utility. 360 regions from the Glasser Human Connectome Project were generated using this procedure (Glasser et al., 2016). 359 regions from this atlas were used for subsequent analyses.

Subcortical and hippocampal segmentation was performed by first assigning one of 39 labels from the FreeSurfer ‘aseg’ feature to each voxel (Fischl et al., 2004b, 2002). 19 of these labels were gray matter structures and were included in subsequent analyses. Additional segmentations of sex-biased nuclei in the hippocampal subfield, amygdala sub-nuclei, and brainstem were made using FreeSurfer joint segmentation of these subfields (Iglesias et al., 2015b, 2015a; Saygin et al., 2017). Segmentations for classically sex-biased BNST and hypothalamic nuclei were made under a different atlas that is not available within FreeSurfer (Neudorfer et al., 2020) (https://zenodo.org/record/3942115). Hypothalamic atlas labels were registered to the study’s average template, and deformation-based morphometry was applied to warp each subject’s image to the study’s template (Devenyi GA, 2024) (https://github.com/CoBrALab/optimized_antsMultivariateTemplateConstruction). The Jacobian determinant from this process was used to calculate the volume change from the template at each voxel in the region of interest (ROI). A summation of these changes within the ROI results in its volume measurement.

### Comparing regional volume covariance between males and females in each species

To compare region of interest (ROI) covariance across sex in each species, we split the data for each species by sex and regressed age and sex out of ROI volumes for both species using the following model:

$$ROI\_volume\;\sim \;intercept+\beta_{1}\;(age)\;+\;Ɛ$$

Residuals from this model were used to compute all pairwise inter-regional volume correlations within males and females. The distributions of these correlations across all pairwise relationships were directly compared between males and females using t-tests and reported 95% confidence intervals (CI). We then subtracted the male correlation matrix from the female to derive a measure of sex differences in correlation for all region pairs (with positive values indicating a larger correlation in males vs. females). The statistical significance of sex differences in correlation for each unique pair of regions was determined by repeating the above process 1000 times with sex being permuted across individuals for each iteration. This procedure yielded a vector of 1000 null values for each pairwise correlation sex differences and we derived empirical p-values for these observed sex differences against these nulls. Empirical p-values were corrected for multiple comparisons across edges using the False Discovery Rate (FDR) (Benjamini and Hochberg, 1995; Benjamini and Yekutieli, 2001) correction with q (the expected proportion of falsely rejected nulls) being set at 0.05.

### Examining the relationship between sex differences in volume covariance and sex differences in regional volume

Sex differences in regional volume were estimated as follows: ROI and total grey matter tissue volumes (TTV) were z-scored across individuals, then input into the following models to estimate the effect of sex on the mean volume of each brain region (given by the $\beta_{1}$ coefficient in the model below):

$$Mice:\;ROI\_volume\;\sim \;intercept\;+\;\beta_{1}(Sex:\;male\;vs\;female)\;+\;\beta_{2}(age)\;+\;\beta_{3}(TTV)\;+\;\beta_{4}(Background\;Strain)\;+\;Ɛ$$

$$Human:\;ROI\_volume\;\sim \;intercept\;+\;\beta_{1}(Sex:\;male\;vs\;female)\;+\;\beta_{2}(age)\;+\;\beta_{3}(TTV)\;+\;\beta_{4}(Euler\;number)\;+\;Ɛ$$

Positive beta coefficients in the model indicate male-biased regional volumes. p-values associated with the $\beta_{1}$ coefficients from each of these models were corrected for multiple comparisons across the number of brain regions in each species using FDR with q < 0.05.

The relationships between covariance and volumetric sex differences were assessed using several complimentary approaches. First, we selected three classically sex-biased regions in mice – the BNST, medial amygdala, and olfactory bulb – and asked if there are any covariance sex differences among these pairings. Statistical significance was calculated using the previously described permutation pipeline and Bonferroni corrected for the total number of comparisons made within this analysis (q = 0.05). Second, we used the full correlation sex differences matrix in each species to estimate the mean sex differences in volume correlation per brain region (averaging the sex differences in its correlation with all other regions) - once using all pairwise correlation sex differences, and again using just those pairs deemed statistically significant in covariance sex differences through permutation testing. We examined the distribution of these properties across the brain of each species and noted those brain regions showing both sex differences in mean volume and sex differences in volume covariance. Third, we used network visualization to specify sets of brain regions showing prominent sex differences in anatomical covariance. Specifically, we identified all region pairs with statistically significant covariance sex differences, converted these pairings into a graph with nodes (regions) and edges (covariance sex differences), and visualized the largest connected components of these graphs in each species to determine their contents and any included nodes that also show sex-biased volume. To capture a similar number of nodes across species for these graphical representations we examined the two largest connected components in mice and the single most connected component in humans. Fourth, we tested if inter-regional variation in the mean sex difference in volume covariance per brain region was correlated with inter-regional variation in the effect size of volumetric sex differences ($\beta_{1}$ coefficients). These correlations were run twice in each species – once using the regional means for the absolute sex differences in correlations and once using regional means for signed sex differences in correlations. The statistical significance of these correlations between regions’ sex differences in covariance and regional sex differences in volume was assessed by comparing observed correlations with a distribution of 1000 null correlations from permutations of sex within species (performed on the NIH HPC Biowulf cluster -- http://hpc.nih.gov).

### R versions and packages

All analyses presented in this paper were performed using R version 4.2.3 unless Biowulf handled the computation, in which case R version 4.2.1 was used (R Core Team, 2023). Packages used for all analyses can be found in the references section (Allen Institute for Brain Science. Allen Brain Explorer., n.d.; Glur, 2020; Kuhn and Wickham, 2020; Landau, 2021; Lander, 2018; Lerch, 2023; Lerch et al., 2017; Mowinckel and Vidal-Piñeiro, 2023, 2022, 2019; Pedersen, 2024; Robinson et al., 2023; Wickham, 2020; Wickham et al., 2019). Data cleaning and analyses codes can be found at github.com/phamlk/cross-species-covariance-sex-differences.

## RESULTS

### Structural covariance is generally stronger in females than males for both mice and humans

For both species, comparing the distributions of all correlations in each sex showed that mean interregional covariance is stronger in females than males, with a small effect size for the mean between sex difference (Δ) in covariance (Figure. 1 A - B, Δ mice: 0.043, 95% CI: 0.041 – 0.045; Δ humans: 0.016, 95% CI: 0.015 – 0.017). After correction for multiple comparisons across region pairs, we identified 44 pairs of regions with statistically significant sex-biased covariance in mice (0.14 % of all pairwise relationships in the mouse brain; 43 stronger covariance in females) and 100 such pairs in humans (0.10 % of all pairwise relationships in the human brain: 71 stronger covariance in females). As expected, the distribution of covariance strengths for these pairs differed between the sexes for both species - with a larger effect size than was seen when considering all pairs (Figure 1 C - F, Δ mice: 0.293, 95% CI: 0.235 – 0.353; Δ humans: 0.156, 95% CI: 0.108 – 0.204). Of note, the average within-sex correlation was consistently higher in mice than in humans (all comparisons: 0.275 in male mice, 0.196 in male humans. 0.318 in female mice, 0.212 in female humans; significant comparisons: 0.129 in male mice, 0.106 in male humans. 0.422 in female mice, 0.261 in female humans). The full within-sex structural covariance matrices and lists of pairwise covariance sex differences can be found for each species in **Extended Figures 1-1**, 1-2 and Extended Tables 1-1, 1-2. As part of these analyses, we also tested the hypothesis that sex differences in volume correlation are more pronounced in region pairs showing weaker within sex volume correlation (as simulated in **Extended Figure 1–3**). We confirmed the expected inverse relationship between covariance strength and covariance sex differences when considering only pairs with statistically significant sex differences in correlations. The results of these analyses are provided in **Extended Figure 1–4**. Taken together these results indicate that regional volume covariance is stronger in mice than humans for both sexes, and that within each species, females tend to show stronger structural covariance than males. For both species, these sex-differences in structural covariance are statistically significant for a small subset (<0.15%) of all possible inter-regional pairings in each species, with the largest sex differences occurring between those inter-regional pairings that show weaker structural covariance in each sex.

### Cross-brain analysis reveals a weak association between interregional sex differences in volume covariance and sex differences in regional volume

We took several complementary approaches to examining the relationship between regional sex differences in brain volume covariance and regional sex differences in brain volume. First, we selected 3 classical regions with the largest and best-replicated sex differences in volume in mice – BNST, olfactory bulb, medial amygdala – and examined sex differences in covariance between these structures. None of the region pairs showed statistically significant sex differences in covariance (Figure 2 B–D: medial amygdala – BNST: Δ = 0.053, p = 1.0; olfactory bulb – BNST: Δ = 0.041, p = 1.0; medial amygdala – olfactory bulb: Δ = 0.083, p = 1.0).

Second, we expanded our analyses to characterize the relationship between sex-biased volume and sex-biased volume covariance throughout the brain of each species more broadly. To contextualize these analyses, we projected all observed sex-differences in volume covariance into anatomical space by computing the mean signed sex-difference in volume covariance for each region and visualizing the distribution of this regional value across the brain of each species (Figure 3 A, B – left columns). Re-computing these maps using information for just those region pairs with statistically significant sex differences in covariance (see Methods) highlighted several brain regions in each species with significant cumulative sex-differences in volume covariance with the rest of the brain (Figure 3 A, B -- right columns). In mice, these regions had almost exclusively stronger covariance in females and included the infralimbic area, medial parietal association cortex, and the pons. Regional covariance sex differences were also more often female-biased in humans and included regions such as the auditory complex 4, the primary sensory cortex, and the primary motor cortex. However, in humans, we also observed regions with significantly male-biased sex-differences in regional structural covariance, including the area prostriata and the premotor eye field. Qualitatively some of the regions highlighted by these analyses also showed sex-differences in mean volume [e.g. mice: mamillary body, mouth primary somatosensory area, claustrum (female-biased mean volume); BNST, olfactory bulb, CA3 pyramidal region (male-biased volume) / humans: primary sensory cortex, cingulate regions (supplementary and cingulate eye field, ventral area 24d) (female-biased volume); hypothalamus, amygdala, posterior insular region 1 (male-biased volume). The full lists of mean regional covariance sex differences in each species can be found in Extended Tables 3-1, 3-2.

To disentangle individual inter-regional pairs from these regional summaries of Figure 3, and to further assess the involvement of volumetrically sex-biased regions in sex-biased covariance patterns, we generated graphs containing all inter-regional pairs with statistically significant sex-biased volume covariance in each species (regions as nodes and sex-biased covariance relationships as edges). Figures 4 and 5 represent the largest connected components of these graphs for mice and humans. In mice, the largest components are centered around the right cuneate nucleus and the left infralimbic area. All connecting covariance sex-biased edges are female-biased. Of the 27 regions involved in these components, 7 volumetrically sex-biased regions are distantly associated with the central nodes. These regions include the male-biased hypothalamus and CA3 pyramidal regions, and the female-biased mamillary body and the cerebellar crus regions. The largest connected component in humans contains 35 regions and is mainly centered around the right posterior insular area 1, a volumetrically male-biased region, and the right parainsular region area 52. Approximately 80% of this component’s edges are female-biased. Only 2 other volumetrically sex-biased regions are represented in this component (supplementary and cingulate eye field; primary sensory cortex – both are female-biased).

Finally, we sought to quantitatively test – within both species - if regional variation in the magnitude of sex-biased volume covariance was related to regional variation in the magnitude of sex-biased mean volume. These analyses were repeated twice within each species - once using absolute values and once using signed values for mean regional sex differences in volume covariance (absolute inter-regional sex differences in covariance for computing regional means and absolute sex differences in volume). The absolute values analysis shows no evidence of an association between regional volumetric and mean covariance sex-bias effect size in both humans and mice (Fig. 6A, B, mice: r = 0.03, p = 0.68; humans: r = 0.13, p = 0.98). However, the signed analyses revealed that both species show a weak yet statistically significant inverse relationship between regional covariance and volumetric sex-bias directions (Fig. 6 C, D, mice: r = −0.19, p = 0.002; humans: r = −0.19; p = 0.001). Specifically, in both species, regions of significantly male-biased volume more often show female-biased volume covariance and vice versa for regions of significantly female-biased volume.

Taken together, these analyses help to localize sex-differences in volume covariance within the brains of humans and mice, and further specify the spatial relationship between this phenomenon and accompanying sex-differences in regional volume. We find several regions of prominently sex-biased volume covariance in each species – highlighting the infralimbic area, medial parietal cortex, and pons in mice and auditory complex 4, the primary sensory cortex, and primary motor cortex in humans. Some of these regions overlap with regional sex-differences in volume – highlighting brain areas that show two forms of sex-biased organization (e.g. mamillary body, mouth primary somatosensory area, claustrum in mouse, hypothalamus, amygdala, and posterior insular region 1 in humans). Although significant sex differences in anatomical covariance are not seen amongst major classical foci of sex-biased volume in the mouse brain, there is a subtle yet significant inverse association between sex differences in volume and volume covariance across both the murine and human brain.

## DISCUSSION

In this study, we utilize sex as a natural experiment to ask whether developmental programming plays a role in structural covariance formation within humans and mice. Our results provide a systematic survey of sex-biased neuroanatomical covariance and the conservation of these features across species. We consider each of our main analyses and findings below.

First, en route to separately estimating sex-differences in structural covariance within humans and mice, we observe that mice show stronger neuroanatomical covariance than humans, regardless of sex. We speculate that this novel observation might reflect the combined action of several inter-species differences. The greater genetic and environmental variability across humans as compared to inbred laboratory mice is likely to translate into weaker coordination of anatomical variation within the human vs. murine brain. Greater neuroanatomical covariation in mice than humans may also track with the gross difference in brain size between species – given that larger vertebrates tend to show greater phenotypic variability (Hallgrímsson and Maiorana, 2000) and the strength of inter-regional covariance is likely to drop off with the greater inter-regional distances within the human compared to the murine brain (He et al., 2007; Yee et al., 2018). The species differences we observe in the strength of overall neuroanatomical covariance could also track with the substantially longer lifespan in humans than mice given evidence of age-related decreases in neuroanatomical covariance within humans (DuPre, E., Spreng, R.N., 2017).

Second, we replicate prior reports of female-biased volume covariance in the human brain (Shi et al., 2023; Wierenga et al., 2022) and reveal for the first time that this phenomenon is also evident in the mouse-brain. This inter-species convergence suggests that female-biased neuroanatomical covariance may be an evolutionarily conserved feature, but further study in other species will be required to verify this. Many previously proposed candidate mechanisms for sex-biased anatomical covariance are unable to parsimoniously account the observation of female-biased covariance in both species. Although sex-differences in overall brain-size would predict stronger anatomical covariation in females vs. males for humans [given larger brain size in males and tendency for anatomical covariance to decreases with increasing organism size (Hallgrímsson and Maiorana, 2000)] – this explanation could not account for sex-biased anatomical covariation in mice given the lack of sex-differences of overall brain size in this species (Guma et al., 2024). Similarly, although greater anatomical variability in male vs. female humans (REF) could explain our observation of stronger anatomical covariation in human females vs. males (if male-biased anatomical variance in humans reflects greater developmental noise that is uncorrelated between regions), this explanation would not apply in mice – which do not show the prominent sex-bias in neuroanatomical variability that is evident in humans (Guma et al., 2024; Wierenga et al., 2022, 2018). The most parsimonious mechanistic hypothesis for our findings would be that the tendency towards female-biased neuroanatomical covariance in both humans and mice reflect a genetic and hormonal aspects of sex that is shared between species and capable of shaping inter-regional anatomical covariance. For example, females of both species are biallelic for X-linked gametologs, whereas males of both species are hemizygous for the X- and Y-member of each gametolog pair. Given this, those regionally specific functional divergences of X- vs. Y-gametologs that have recently been reported within the brain (DeCasien et al., 2024) represent a male-specific source of inter-regional divergence in neurobiological organization that is operative in both species and could lead to stronger inter-regional covariance in femalesClick or tap here to enter text..

Third, we profile the regional distribution of sex-biased volume covariance in each species and probe how this are related to much more extensively studied sex differences in mean regional volume. As an initial targeted test for the idea of coordinated sex-differences in brain volume and volume covariance – we focused on 3 regions of canonically sex-biased volume in the mouse brain (BNST, medial amygdala, olfactory bulb) and found no evidence of sex-biased covariance amongst these regions. Thus, those male-specific processes that are documented to drive the highly reproducible male-bias in the mean volume of these regions (Forger et al., 2004; Gorski et al., 1978; Hines et al., 1992; Roos et al., 1988; Segovia et al., 1984) do not appear to open male-specific sources of covariation between regions. However, we cannot rule out the possibility that these regions show sex-specific sources of volume covariation that counterbalance each other or are hidden by dominantly shared sourced of inter-regional volume covariation between the sexes.

While no covariance sex differences were identified among classical regions of volumetric sex bias, broadening the search for sex-biased volume covariance does identify multiple brain regions that show differential volume coupling with the rest of the brain between sexes. Echoing the female-bias in volume covariance found in our global analyses, regional hotspots of sex-biased covariance were almost exclusively female-biased in mice and mostly female-biased in humans. Regions with significant sex-bias in covariance with other regions in mice were the infralimbic area, medial parietal association cortex, and the pons (stronger in females); in humans, these regions include the auditory complex 4, the primary sensory cortex, the primary motor cortex (stronger in females), the area prostriata, and the premotor eye field (stronger in males). Knowing regions of sex-biased volume covariance provides a powerful starting point for probing potential mechanistic relationships between sex-biased anatomical covariance and sex differences in connectivity, function and coordinated developmental influences. Moreover, some of the brain regions showing prominent sex-biased covariance are also volumetric sex-biased in our study, such as the mamillary body and claustrum in mice and the posterior insular region 1 and hypothalamus in humans. These brain regions offer high-priority targets for follow-up mechanistic analyses and for probing how sex-biased anatomical organization of the brain might relate to brain function.

To dissect out specific pairwise sex differences in covariance that underpin these regional patterns, we visualized networks of significant covariance sex differences in mice and humans to flag large sets of brain regions that are all interlinked through sex-biased volume covariance. These findings help to motivate and focus those challenging experimental studies that will now be needed to determine the drivers for sex-differences in neuroanatomical covariance.

While we define some instances of overlap between sex-biased volume covariance and sex-biased volume, we show that these features of the brain are not strongly related across the brain as a whole. This dissociation implies that the biological process regulating development of sex-biased volume are largely dissociable from those that drive sex-biased volume covariance. However, we do observe a weak negative correlation between signed sex differences in brain volume and brain volume covariance – mostly driven by a tendency for regions of male-biased volume to show female-biased volume covariance. Thus, we speculate that the presence of male-specific influences on the size of a sex-biased region introduces male-specific sourced of regional volume variation that are poorly coordinated between regions – resulting in weakened interregional correlations amongst these regions in males as compared to females.

Our findings should be considered with several caveats and limitations. First – we have focused on brain volume as an anatomical phenotype that can be comparably estimated by structural MRI across both species, but there are many other anatomical properties of the brain beyond volume (e.g., regional myelin content). We cannot assume that our volumetric findings here will generalize to other properties of the brain. Second, we estimate covariance cross-sectionally in datasets from adulthood in each species. As such, our study cannot speak to potential age-related variations of sex-biased anatomical covariance. Third, our study design is purely observational, and we cannot dissect the potential mechanistic basis for observed sex differences in covariance. In mice, these mechanisms could ultimately be related to sex-biased chromosomal and gonadal influences on brain development. Such influences are also likely to be operative in humans but will almost certainly be interwoven with co-occurring gendered influences stemming from the external environment. Transgenic models like the Four Core Genotypes could explain how covariance sex differences are formed (Arnold and Chen, 2009; Corre et al., 2016). These experiments would expand our understanding of how sex affects brain organization and how developmental programming, in general, can drive covariance formation.

Notwithstanding the limitations above, our study was able to use the natural experiment of sex to provide evidence for the involvement of developmental mechanisms in brain structural covariance formation. We validate existing human results that show females have stronger covariance and identified that the same phenotype is also present in mice. We provide a fine-grained delineation of those brain systems that show sex-biased covariance in each species. Our finding that structural covariance sex differences only partially involve volumetrically sex-biased structures supports the viewpoint that the influences of sex likely have uncoordinated effects across brain regions and highlights structural covariance as a novel axis of sex-biased brain organization warranting further study. Additionally, the cross-species approach used here allows us to articulate the potential impacts of stochasticity and variation on structural covariance, thus emphasizing the importance of applying synchronized methodologies across species for gaining new insights into brain biology and increasing the translatability of animal findings (Barron et al., 2021).

## Extended Figures

**Figure 7–1.**
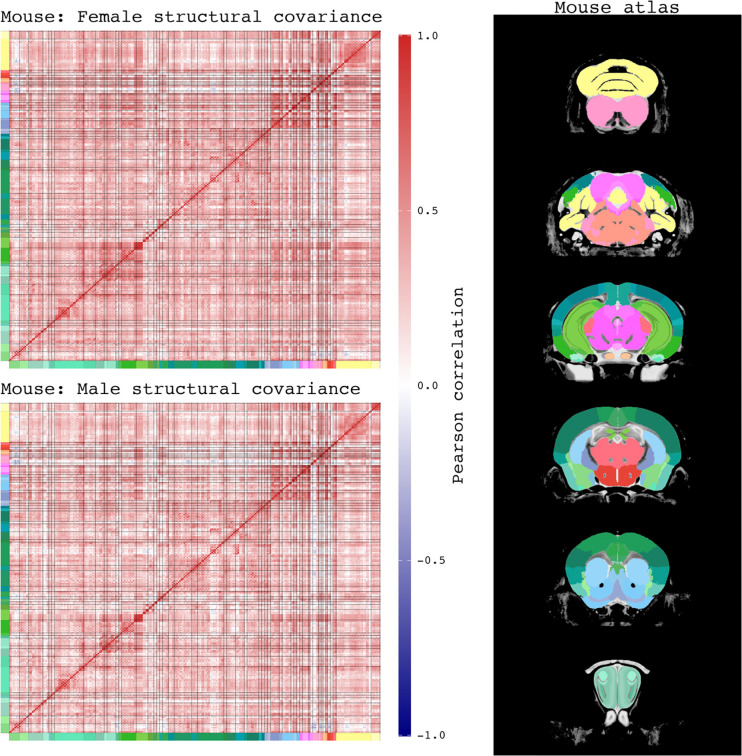
Male and female structural covariance of mouse. Pearson correlation matrices of regional brain volumes in mice, separated by sex. Each row and column represent a grey matter structure defined by the Allen Mouse Brain Atlas, shown in the right column. Structures are denoted by color bands that correspond to their structures’ colors in the atlas. Each element in the matrices represent a Pearson correlation between two brain structures. Mouse brain structures mainly have positive correlations to each other. Given the same correlation color scale across sex, female mice appear to have more intense positive correlations in the brain comparing to males.

**Figure 1–2.**
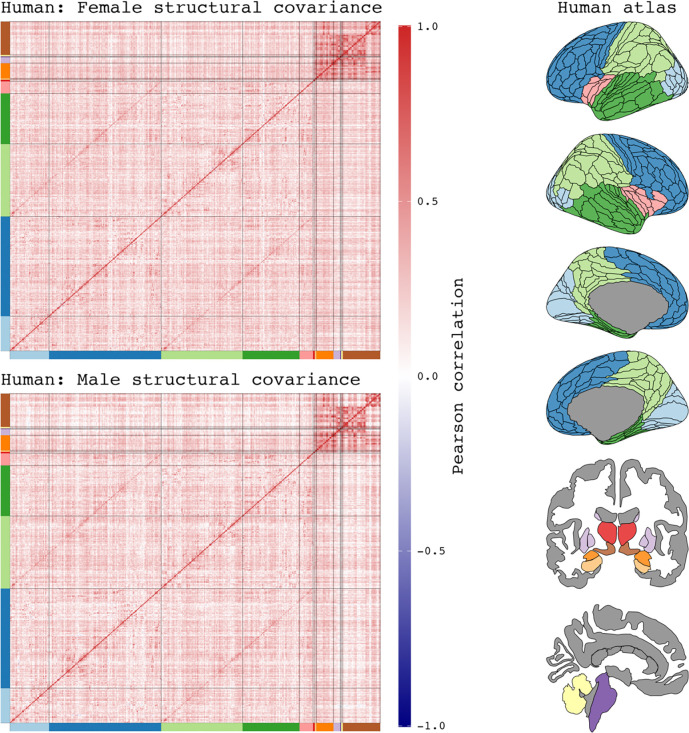
Male and female structural covariance of human. Pearson correlation matrices of regional brain volumes in humans, separated by sex. Cortical structures are grouped into 6 lobes, as defined by Freesurfer output for Glasser atlas segmentation: occipital (light blue), frontal (dark blue), parietal (light green), temporal (dark green), insula (salmon). Subcortical structures are defined as followed using Freesurfer output for Aseg atlas segmentation: thalamus proper (red), amygdala (orange), basal ganglia – combination of caudate, putamen, pallidum (light purple), pons (dark purple), cerebellum (yellow), ventral diencephalon (brown). Additional segmentations of amygdala and hypothalamic nuclei were grouped under the amygdala and ventral diencephalon categories, respectively. Structures are denoted by color bands that correspond to their structures’ colors in the atlas. Each element in the matrices represent a Pearson correlation between two brain structures. Human brain structures mainly have positive correlations to each other. Given the same correlation color scale across sex, females appear to have more intense positive correlations in the brain comparing to males. The correlation color intensities are less than those observed in both male and female mice.

**Figure 1–8.**
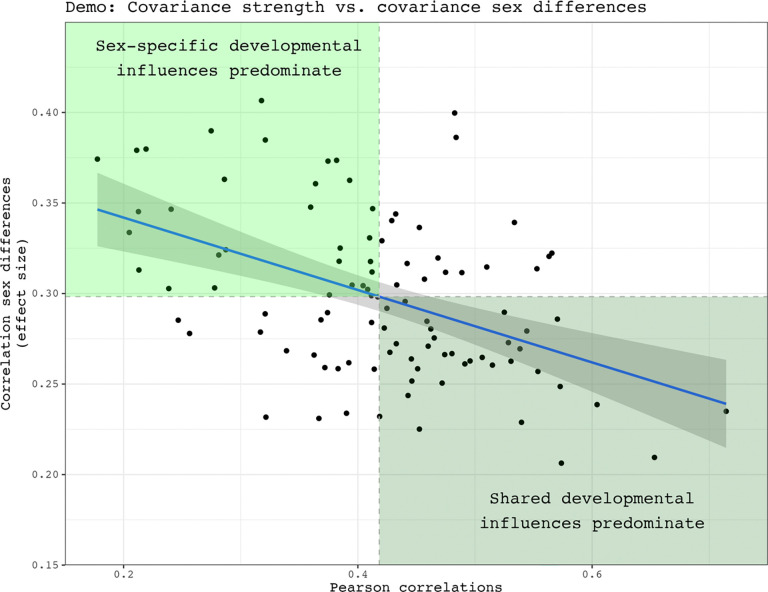
Simulated data demonstration: Shared versus sex-specific developmental influences on structural covariance sex differences. The correlation strength between two structures tend to increase as a function of shared developmental influences, such as through shared axonal connectivity or gene expressions. As structures share less influences, their correlations also tend to weaken. If sex-specific influences only act on certain structures in the brain, then they are more likely to influence the covariance between pairs where one structure receives the developmental influences of sex while the other does not. In other words, sex-specific developmental influences are more likely to act upon structure pairs with less shared influences to each other, or pairs with weaker correlations. For this reason, one could expect covariance sex differences to be largest between covariance pairs with weak associations to each other.

**Figure 1–9.**
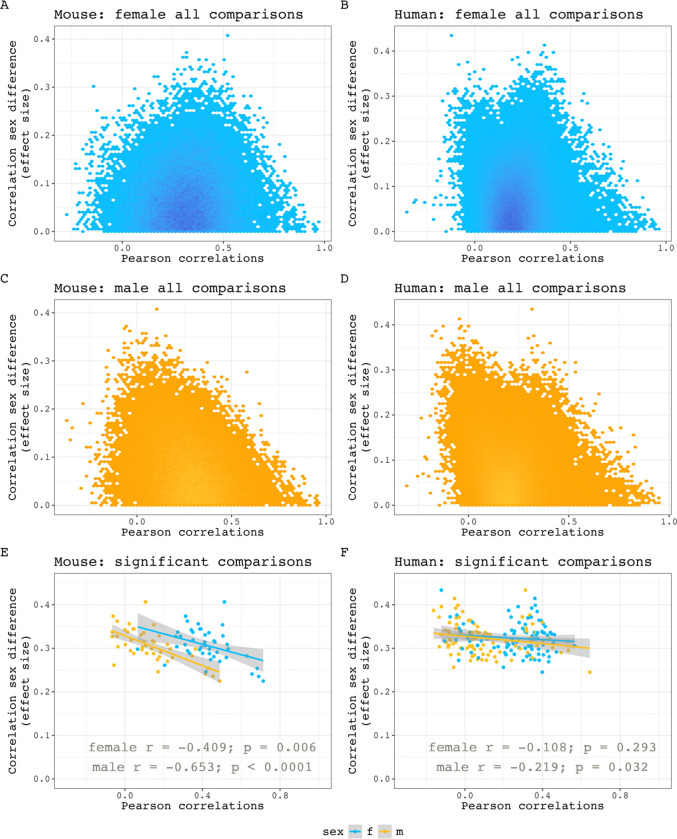
Structural covariance strength versus sex difference in mice and humans. **A-D)** Pairwise Pearson correlations versus absolute correlation sex differences for all comparisons in female mouse (A) and human (B) and male mouse (C) and human (D)**E, F)** Pairwise Pearson correlations versus absolute correlation sex differences in pairs with significant covariance sex differences for mouse (E) and human (F). Association strengths between these values are calculated using Pearson correlation and p-values generated by the cor.test function in R. The predicted inverse relationship between covariance strength and sex differences are more prominent in mice than humans.

## Supplementary Material

Supplement 1 **Extended Table 3-2.** All regional mean covariance sex differences in humans. Signed values are indicated as “meanCov_signed”. Absolute values are indicated as “meanCov_absolute”.

Supplement 2 **Extended Table 3-1.** All regional mean covariance sex differences in mice. Signed values are indicated as “meanCov_signed”. Absolute values are indicated as “meanCov_absolute”.

Supplement 3 **Extended Table 1-2.** All pairwise covariance sex difference results in humans. Differences significance status is defined in the “signif” and “signif_adj” columns, corresponding to whether the difference is significant after permutation testing or after permutation testing and multiple comparisons corrections.

Supplement 4 **Extended Table 1-1.** All pairwise covariance sex difference results in mice. Differences significance status is defined in the “signif” and “signif_adj” columns, corresponding to whether the difference is significant after permutation testing or after permutation testing and multiple comparisons corrections.

## Figures and Tables

**Figure 1.**
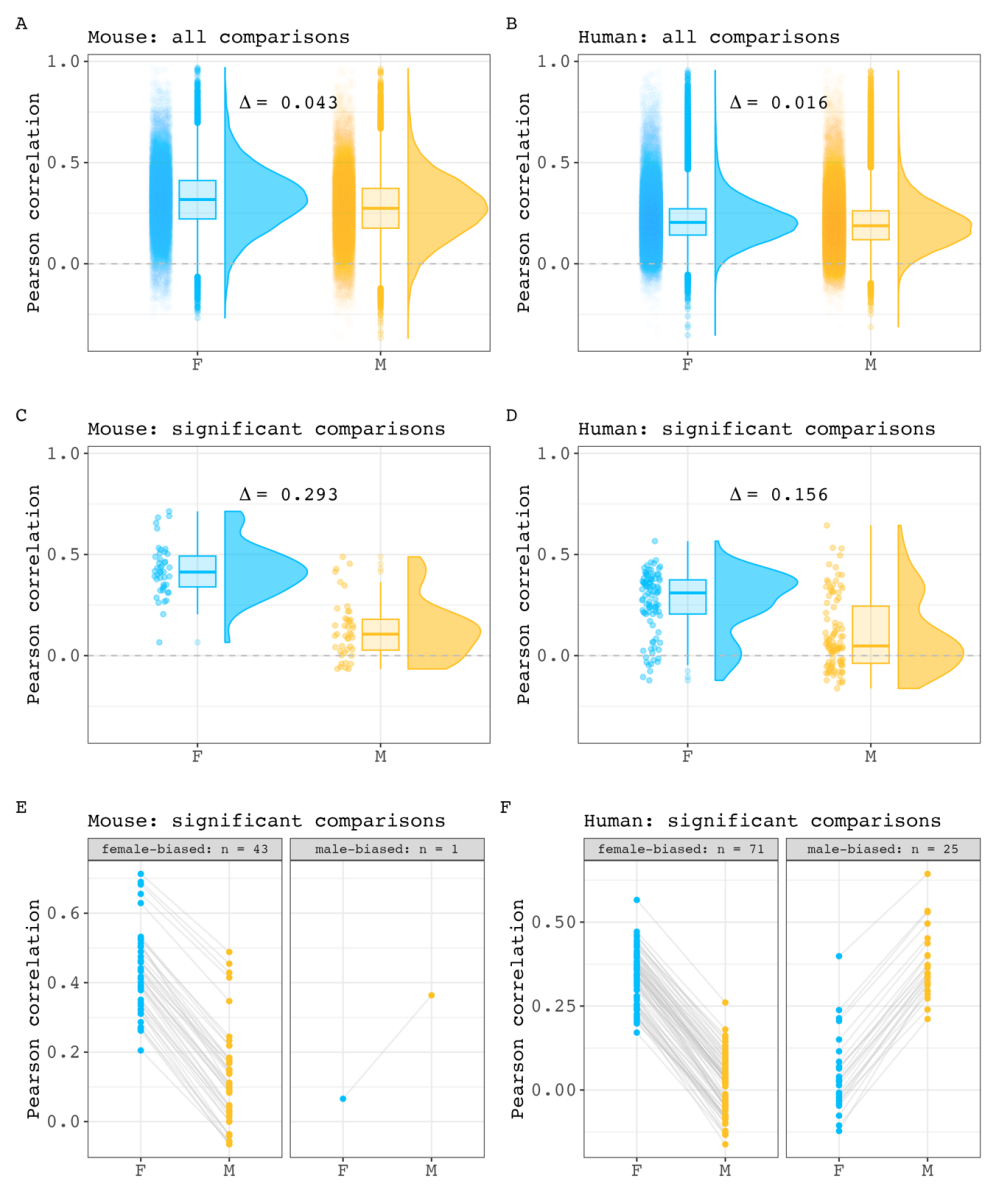
Inter-regional volume correlation distributions across sex in mice and humans. **A, B)** Comparison of all within sex correlation values for mouse (A) and human (B) (Δ mouse = 0.043, 95% CI: 0.041 – 0.045. Δ human = 0.016, 95% CI: 0.015 – 0.017). Each point represents a pairwise correlation value. Box plots and density plots are shown for distribution visualizations. **C, D)** Comparison of within-sex correlation values for region pairings with statistically significant covariance sex differences in mouse (C) and human (D) (Δ mouse = 0.293, 95% CI: 0.235 – 0.353. Δ human = 0.156, 95% CI: 0.108 – 0.204). **E, F)** Pairwise visualization of comparisons with significant sex differences in mouse (E) and human (F). Each point represents a pairwise correlation in either male or female. A connecting line between two points are shown to connect a pairwise correlation value in one sex and with its equivalent pairing in the other sex.

**Figure 2.**
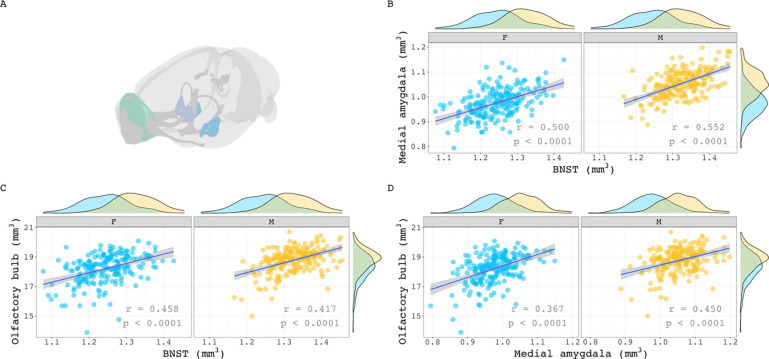
Within sex correlations between classic sex-biased regions in the mouse. **(A)** Visualization of three volumetrically sex-biased regions: olfactory bulb, BNST, and medial amygdala. **B, C, D)** Sex-specific correlations of the medial amygdala with BNST (B), olfactory bulb with BNST (C), and olfactory bulb with medial amygdala (D). Sex-specific means of each region were added to their residuals before correlation calculations began. This was done to maintain volumetric sex differences in volume distribution for visualization purposes. Correlation sex differences for all three pairs were not statistically significant (p = 1.0).

**Figure 3.**
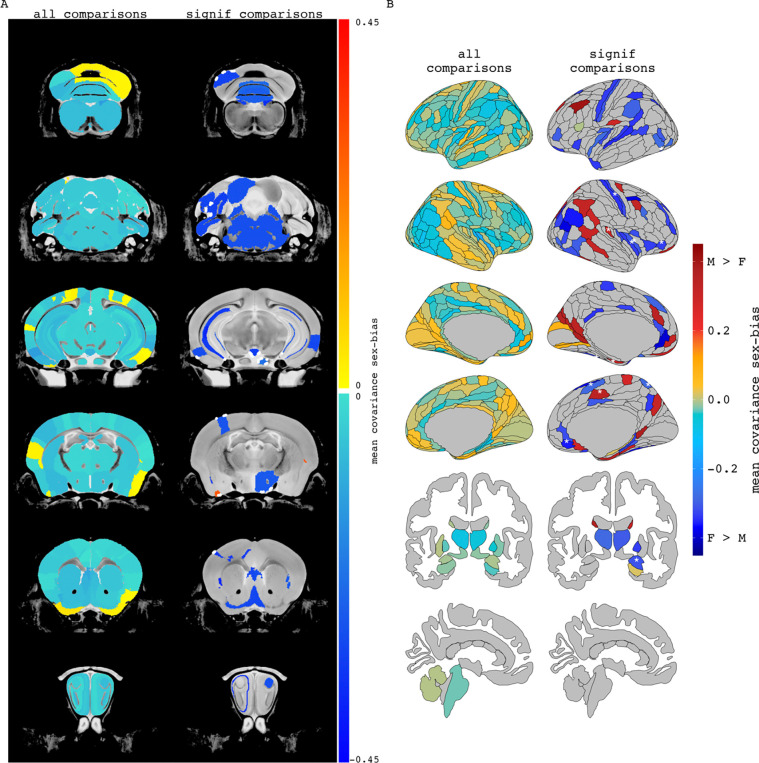
Regional mean covariance sex differences in mice and humans. Mean covariance sex differences in mouse (A) and human (B) when averaging a region’s covariance sex difference across all pairwise comparisons (all comparisons) or only pairwise comparisons with statistically significant covariance sex differences (significant comparisons). When including all pairwise comparisons, 89% of mouse regions had mean covariance sex differences which were stronger in females. For humans, averaging all pairwise covariance sex differences per brain region resulted in 68% of human regions with female-biased mean covariance. When including only comparisons significant for covariance sex differences, 96% of the included mouse regions and 70% of the human regions were female biased. Regions containing statistically significant covariance sex differences that are also sex-biased in volume are highlighted by dashed lines in the mouse brain and asterisks in the human brain.

**Figure 4.**
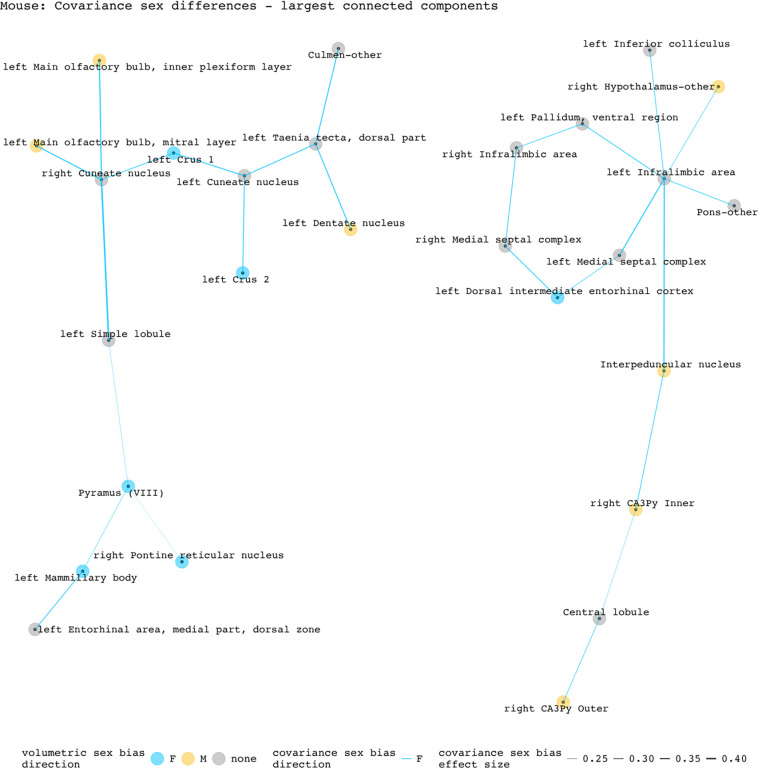
Statistically significant covariance sex differences in mice, represented as nodes and edges (top 2 most connected components). The first component (left side) is centered by the right cuneate nucleus and involves 14 regions/13 covariance pairs. The second component (right side) is centered on the left infralimbic area and involves 13 regions/13 covariance pairs. Both components contain only female-biased edges. Of the 16 edges involving volumetrically sex-biased regions in these components, 13 are between a volumetrically sex-biased region and a region without any volumetric sex bias; 3 are between two volumetrically sex-biased regions.

**Figure 5.**
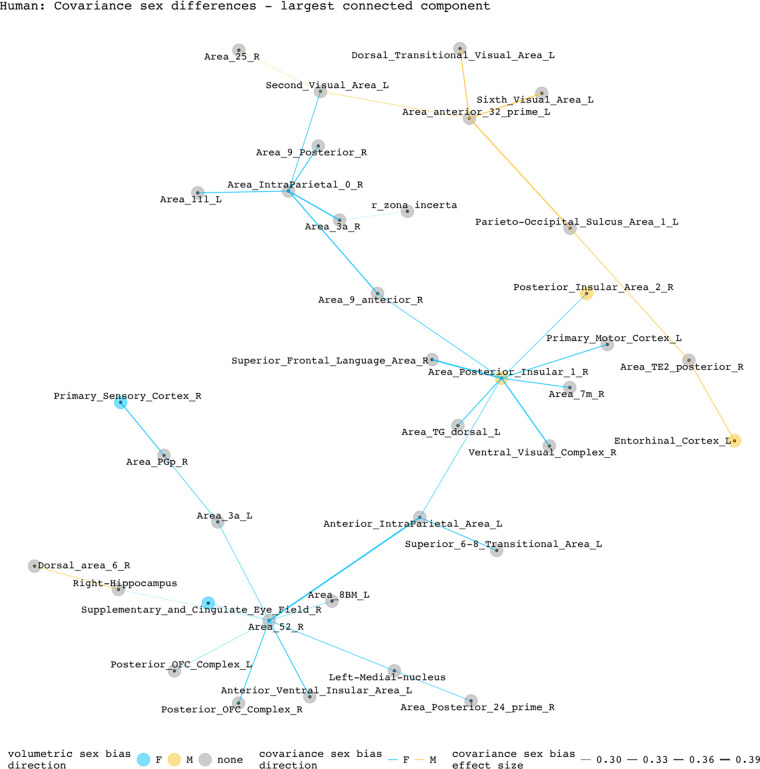
Statistically significant covariance sex differences in humans, represented as nodes and edges (most connected component). This component is centered by two regions: right parainsular area 52 with 7 connecting nodes and right posterior insular area 1 with 8 connecting nodes. Of the 33 edges included in this component, 8 are male-biased. Of the 35 regions included in this component, 3 have volumetric sex differences (right posterior insular area 1, right posterior insular area 2, right supplementary and cingulate eye field). Except for the female-biased covariance relationship between the right posterior insular area 1 and the posterior insular area 2, all sex-biased edges involving a volumetrically sex-biased region (9 in total) are paired with a volumetrically non-sex-biased region.

**Figure 6.**
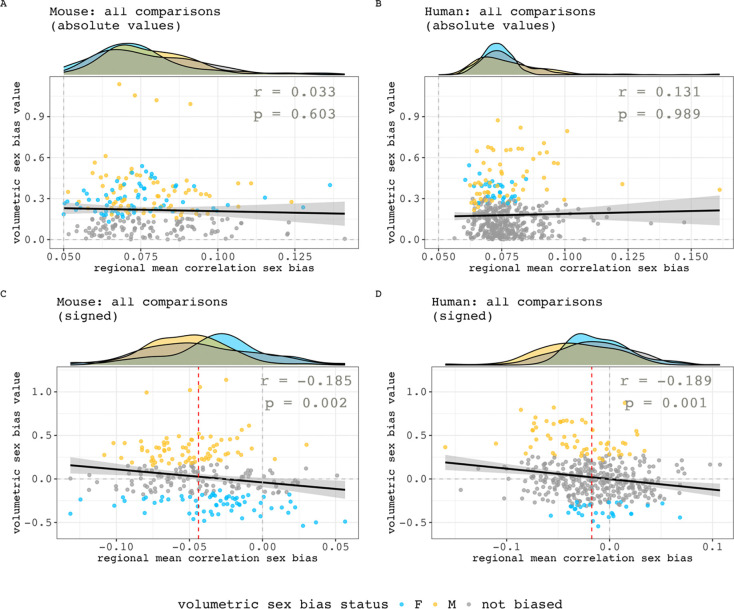
Relationships between the sex-biased in regional volumetric and regional volume covariance. **A, B)** Relationships between absolute values in mean regional correlation sex bias and volumetric sex biases in mouse (A) and human (B). **C, D**) Relationships between signed values in mean regional correlation sex bias and volumetric sex biases in mouse (C) and human (D). Negative values are female biased in both axes. Marginal density plots represent the regional mean correlation sex-bias distributions of different volumetric sex-bias categories (female-biased, male-biased and not significantly sex-biased). For the signed analyses in both species, there is a statistically significant, but weak negative correlation between sex-differences in volume and sex-differences in volume covariance (inset statistics). Thus - in both species - regions of significantly male-biased volume more often show female-biased volume covariance and vice versa for regions of significantly female-biased volume. However, note also that regions with the largest volumetric sex-bias values do not have the largest mean correlation sex-bias – their values are concentrated closer to the median regional mean correlation sex bias (red dashed line). Also, regions with statistically significant volumetric sex biases but smaller volumetric sex bias values can be found on either end of the x-axis.

**Table 1:** 

	Female	Male	Statistics
**Sample size**	211	212	
**Age (days)**			
Mean	62.0	62.6	F (1, 421) = 0.49, p = 0.44
SD	7.6	8.5	
Range	56 – 90	56 – 90	
**Background Strain**			
C57BL-6J (12 cohorts)	132	139	X^2^ = 0.29, p = 0.59
C57BL-6N (6 cohorts)	79	73	

**Table 2:** 

		Females	Males	Statistics
**Sample size**		238	198	F (1, 434) =25.99, p = 5.143e-07
**Age**	*Mean*	29.4	27.6	
	*SD*	3.7	3.6	
	*Range*	22 – 36	22 – 35	
**Education (in years)** *	*Mean*	15.0	14.8	F (1,433) =1.45, p=0.23
	*SD*	1.8	1.7	
	*Range*	11 – 17	11 – 17	
**Euler number**	*Mean*	−53.7	−59.0	F (1,434) = 8.29 p=0.004
	*SD*	18.4	20.6	
	*Range*	−126 to −16	−201 to −20	
**Zygosity** *	*Monozygotic*	72	37	X^2^ = 10.84, p = 0.004
	*Dizygotic*	66	49	
	*Not Twin*	99	111	

* 1 subject did not have education information reported. 2 subjects did not have zygosity information reported. Statistics were only performed on subjects with available demographic data.

** ANOVA and chi-square tests of significant difference between groups (males vs. females). SD = standard deviation.

## SIGNIFICANCE STATEMENT

Structural covariance is a potent readout of coordinated brain development, but hard to probe experimentally. Here we use sex differences as a naturally occurring test for developmental theories of structural covariance – adopting a cross-species approach for validation and translational benefit. Brain MRI reveals two conserved features of anatomical covariance across humans and mice: (i) tighter inter-regional coordination of brain development in females as evidenced by stronger volume covariance; (ii) a tendency for female-biased covariance to involve regions that are smaller in females – suggesting an unknown counterbalancing between these two distinct modes of sex-biased brain organization. These findings advance understanding of coordinated brain development and sex difference in a cross-species framework – facilitating future translational research on both topics.
