# Supplementary figures and images for: A cross-species analysis of neuroanatomical covariance sex differences in humans and mice
Source: bioRxiv. 2025 Apr 8:2024.11.05.622111. Originally published 2024 Nov 5. Preprint. [Version 2] doi: 10.1101/2024.11.05.622111 (PMC11580902; doi:10.1101/2024.11.05.622111)

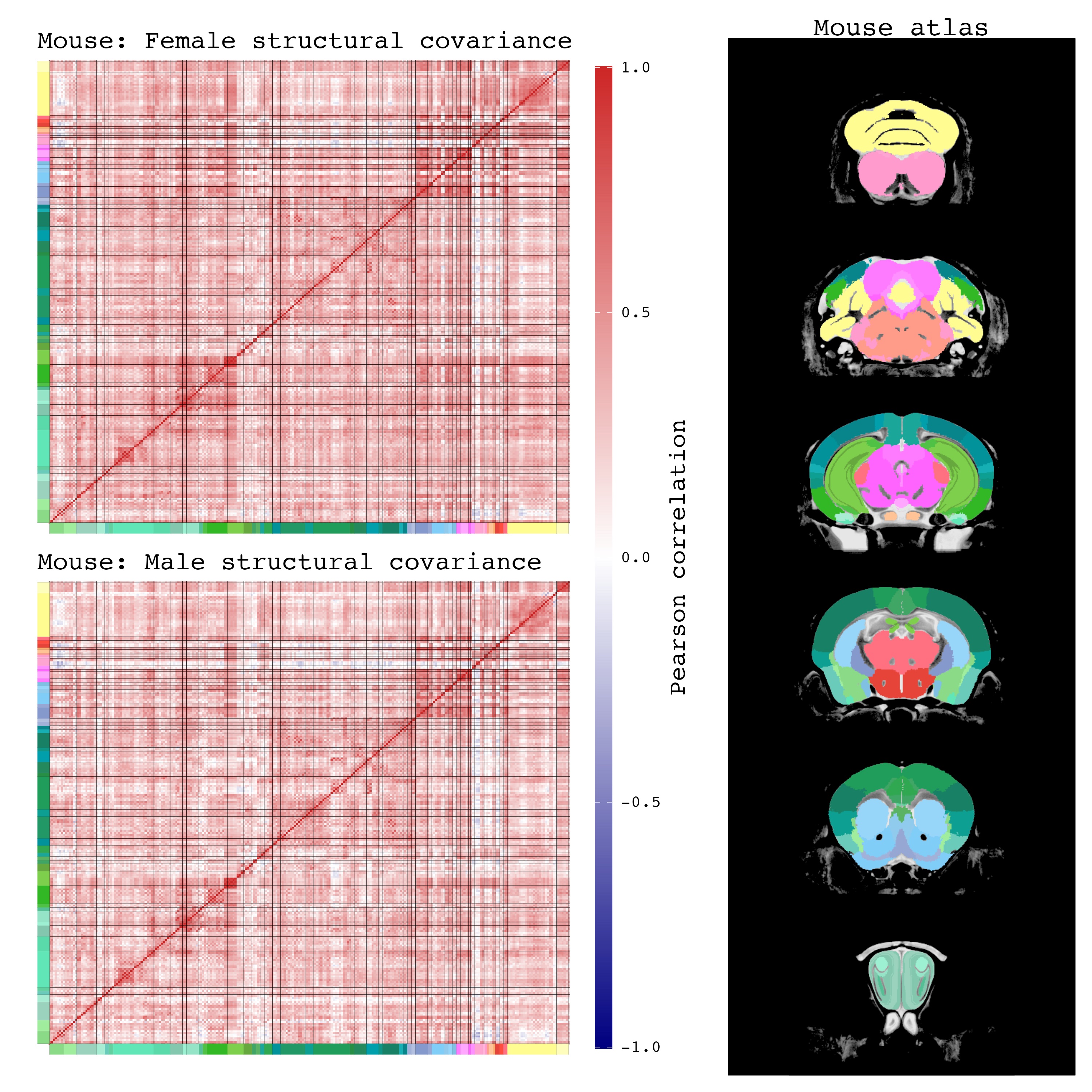

Supplement: Supplement 5 — Figure 1-1. Male and female structural covariance of mouse. Pearson correlation matrices of regional brain volumes in mice, separated by sex. Each row and column represent a grey matter structure defined by the Allen Mouse Brain Atlas, shown in the right column. Structures are denoted by color bands that correspond to their structures’ colors in the atlas. Each element in the matrices represent a Pearson correlation between two brain structures. Mouse brain structures mainly have positive correlations to each other. Given the same correlation color scale across sex, female mice appear to have more intense positive correlations in the brain comparing to males. [file media-5.jpg]

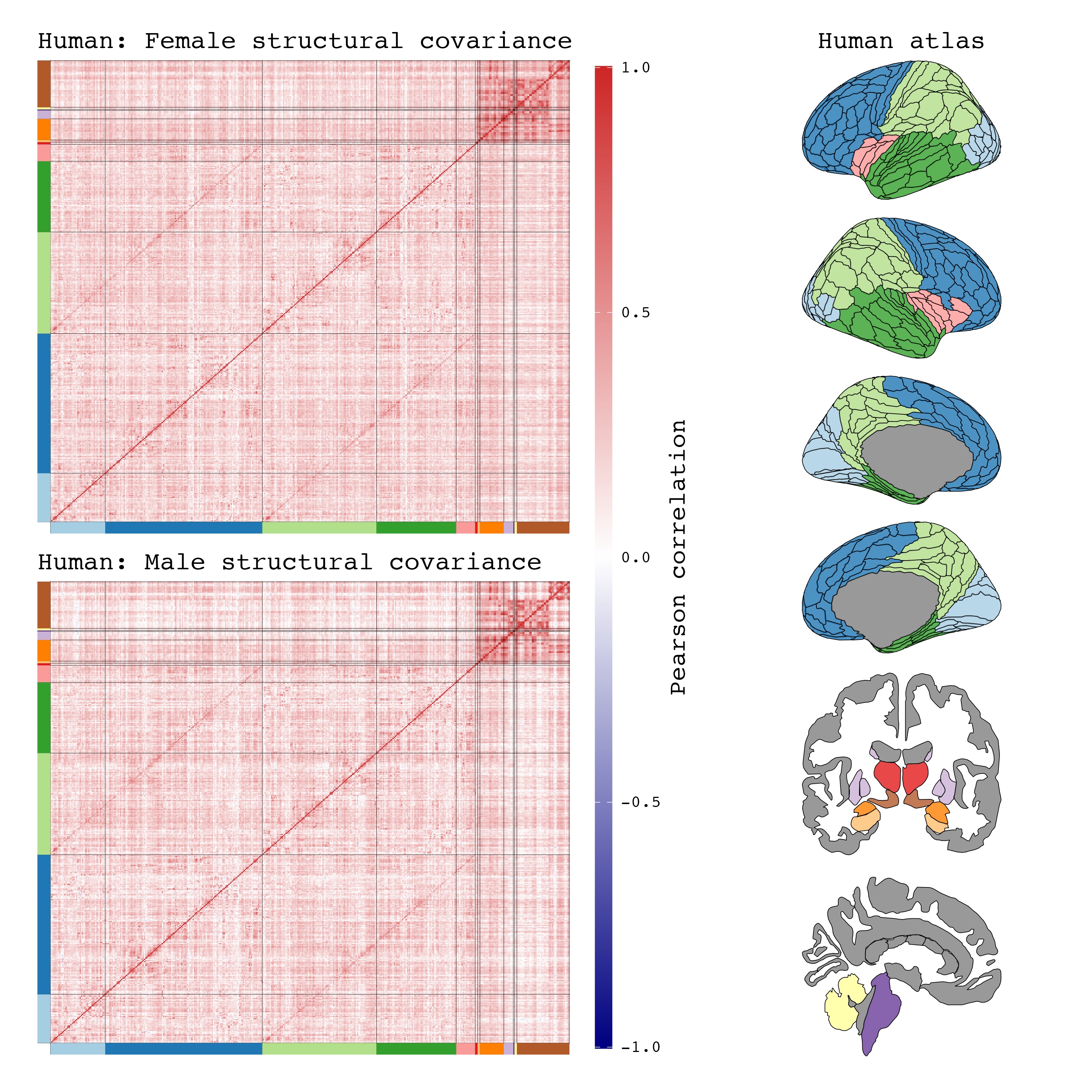

Supplement: Supplement 6 — Figure 1-2. Male and female structural covariance of human. Pearson correlation matrices of regional brain volumes in humans, separated by sex. Cortical structures are grouped into 6 lobes, as defined by Freesurfer output for Glasser atlas segmentation: occipital (light blue), frontal (dark blue), parietal (light green), temporal (dark green), insula (salmon). Subcortical structures are defined as followed using Freesurfer output for aseg atlas segmentation: thalamus proper (red), amygdala (orange), basal ganglia – combination of caudate, putamen, pallidum (light purple), pons (dark purple), cerebellum (yellow), ventral diencephalon (brown). Additional segmentations of amygdala and hypothalamic nuclei were grouped under the amygdala and ventral diencephalon categories, respectively. Structures are denoted by color bands that correspond to their structures’ colors in the atlas. Each element in the matrices represenst a Pearson correlation between two brain structures. Human brain structures mainly have positive correlations to each other. Given the same correlation color scale across sex, females appear to have more intense positive correlations in the brain comparing to males. The correlation color intensities are less than those observed in both male and female mice [file media-6.jpg]

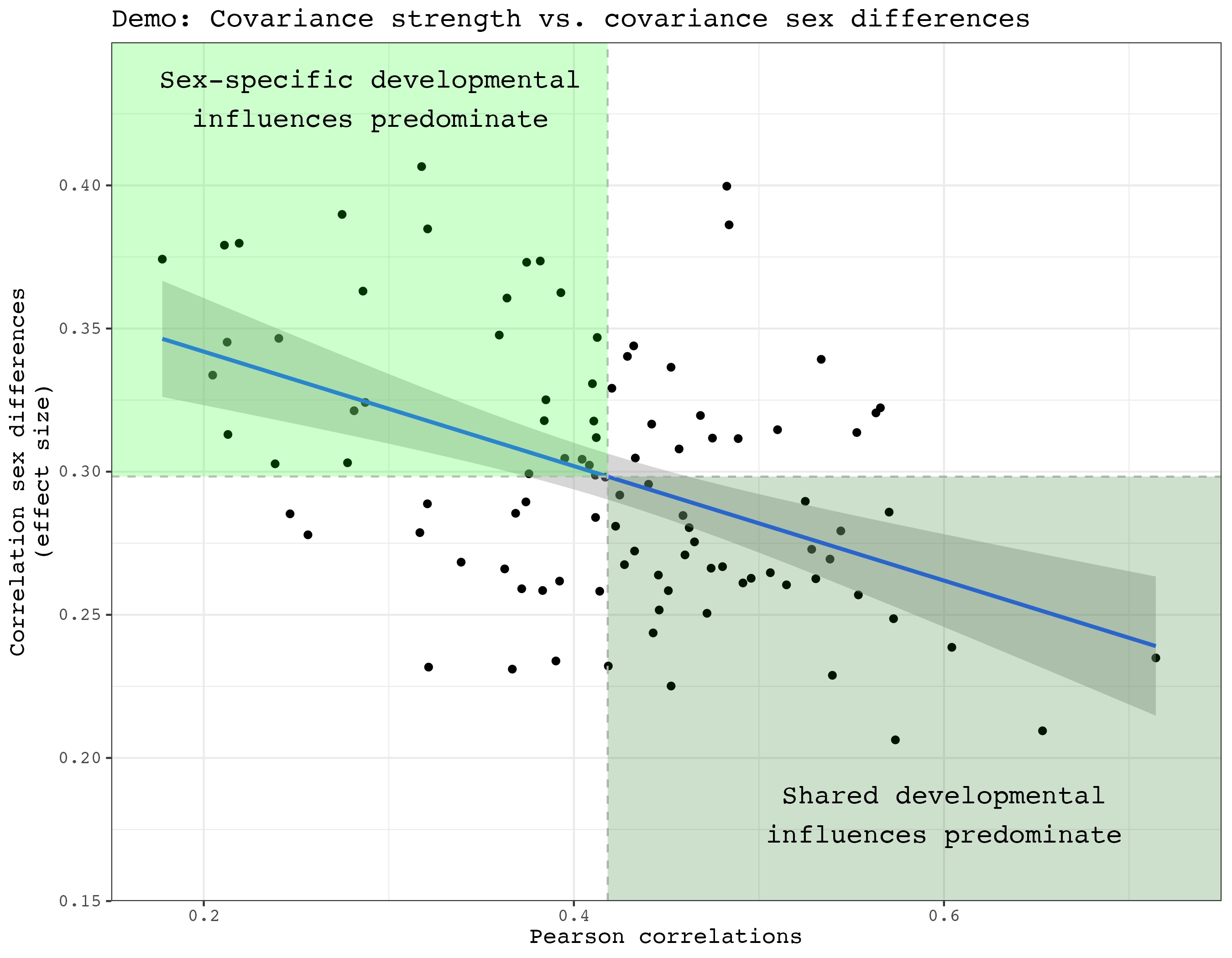

Supplement: Supplement 7 — Figure 1-3. Simulated data demonstration: Shared versus sex-specific developmental influences on structural covariance sex differences. The correlation strength between two structures tends to increase as a function of shared developmental influences, such as through shared axonal connectivity or gene expressions. As structures share less influences, their correlations also tend to weaken. If sex-specific influences only act on certain structures in the brain, then they are more likely to influence the covariance between pairs where one structure receives the developmental influences of sex while the other does not. In other words, sex-specific developmental influences are more likely to act upon structure pairs with less shared influences to each other, or pairs with weaker correlations. For this reason, one could expect covariance sex differences to be largest between covariance pairs with weak associations to each other [file media-7.jpg]

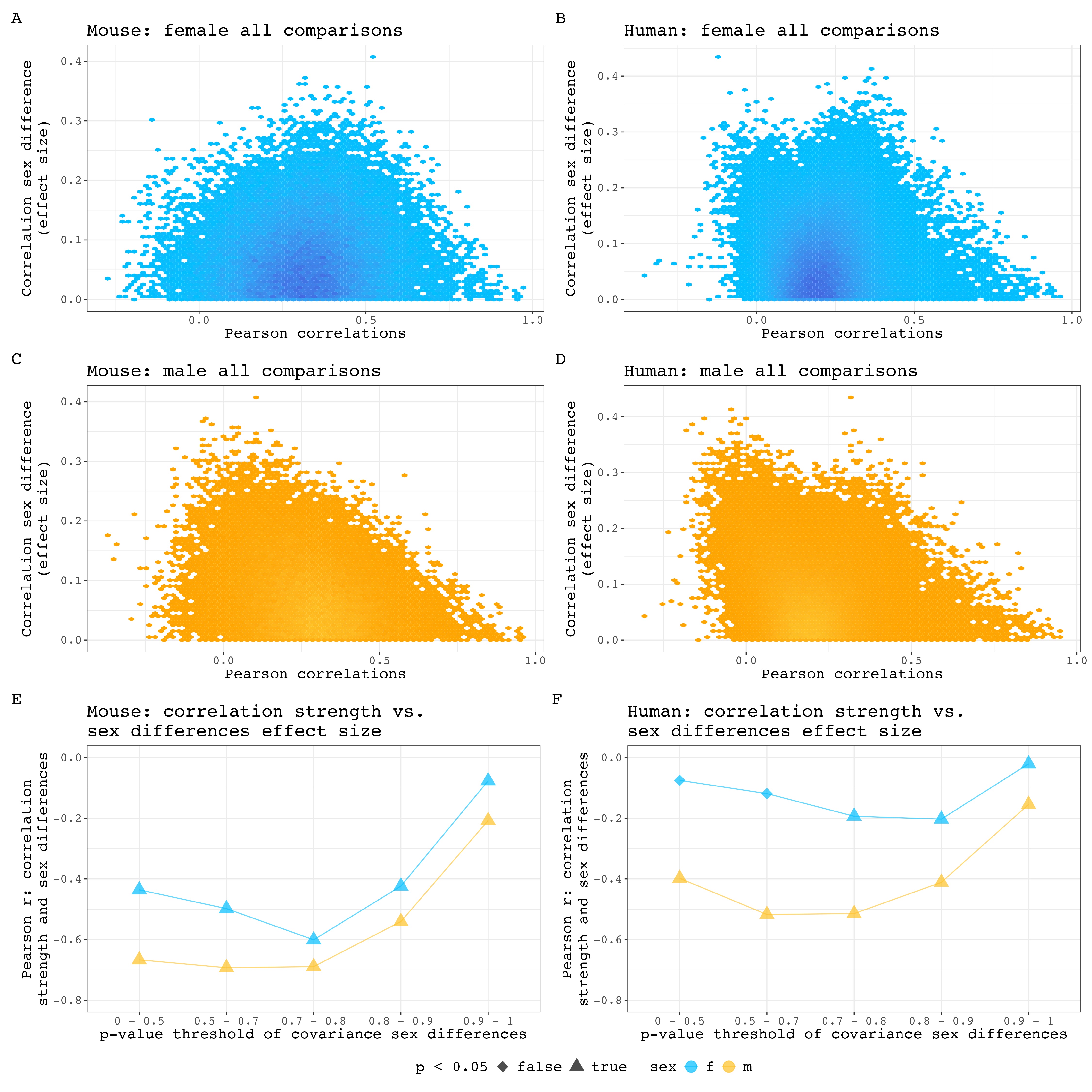

Supplement: Supplement 8 — Figure 1-4. Structural covariance strength versus sex difference in mice and humans. A-D) Pairwise Pearson correlations versus absolute correlation sex differences for all comparisons in female mouse (A) and human (B) and male mouse (C) and human (D). E, F) Pairwise Pearson correlations versus absolute correlation sex differences at different covariance sex differences significance thresholds for mouse (E) and human (F). Association strengths between the covariance sex differences of significant pairs and their Pearson correlations are calculated using Pearson correlation and p-values generated by the cor.test function in R. The predicted inverse relationship between covariance strength and sex differences are more prominent in mice than humans. [file media-8.jpg]
